# Bratter: An Instruction Set Extension for Forward Control-Flow Integrity in RISC-V

**DOI:** 10.3390/s22041392

**Published:** 2022-02-11

**Authors:** Seonghwan Park, Dongwook Kang, Jeonghwan Kang, Donghyun Kwon

**Affiliations:** 1 Computer Security Laboratory, School of Computer Science & Engineering, Pusan National University, Busan 609-735, Korea; starjara@pusan.ac.kr (S.P.); jeonghwan@pusan.ac.kr (J.K.); 2 Cyber Security Research Division, Electronics and Telecommunications Research Institute, Daejeon 305-700, Korea; dkang@etri.re.kr

**Keywords:** CFI, RISC-V, ISA

## Abstract

In recent decades, there has been an increasing number of studies on control flow integrity (CFI), particularly those implementing hardware-assisted CFI solutions that utilize a special instruction set extension. More recently, ARM and Intel, which are prominent processor architectures, also announced instruction set extensions for CFI called branch target identification (BTI) and control-flow enhancement technology (CET), respectively. However, according to our preliminary analysis, they do not support various CFI solutions in an efficient and scalable manner. In this study, we propose Bratter, a new instruction set extension for forward CFI solutions on RISC-V. At the center of Bratter, there are *Branch Tag Registers* and dedicated instructions for these registers. We implemented well-known CFI solutions (i.e., branch regulation and function signature check) using Bratter to evaluate its performance. Our experimental results show that, by using Bratter, even when these two solutions work together, they impose only 1.20% and 5.99% overhead for code size and execution time, respectively.

## 1. Introduction

In recent decades, control-flow integrity (CFI) has been considered to effectively prevent a number of control hijacking attacks such as return-oriented programming (ROP) and jump-oriented programming (JOP) attacks. Basically, CFI thwarts these attacks by checking whether the target address of control transfer instruction (CTI) is legitimate before executing the instruction. CFI solutions can be categorized into forward CFI for indirect call/jump and backward CFI for return. In general, it is known that backward CFI can be safely guaranteed by using shadow stack [1], however, in the case of the forward CFI, various methods have been conducted. For example, to check whether the target address of indirect calls is valid, some studies have utilized a control flow graph (CFG) [2] whereas others make use of the function type [3].

To implement such various policies, some researchers have proposed a software-based approach in which additional instructions are inserted to every CTI to prevent control hijacking attacks exploiting the instruction. As the added instructions consist of a couple of arithmetic and memory instructions, this approach is applicable regardless of the processor architecture and software type (e.g., OS kernel and user application). However, the added instructions promote considerable performance overhead. Thus, to relieve such overhead, several commodity processor architectures introduced instruction set extensions for CFI, such as ARM branch target identification (ARM BTI) [4] and Intel CETS [5]. By enforcing CFI with special instructions, the amount of added instructions for check operation can be reduced and the performance can be improved.

However, according to our preliminary analysis, these extensions do not provide sufficient programmability to implement various CFI policies. In short, BTI and CETS only enforce that the instruction after executing indirect CTIs (i.e., indirect call and indirect jump) should be the special instruction (bti in ARM BTI and endbranch in Intel CETS). Thus, they cannot be used to implement finer-grained CFI solutions that enforce different rules for every indirect CTI. Although researchers [6,7,8,9,10] have proposed an extension that can define multiple rules to address this issue, it cannot efficiently support context-sensitive rules. More details are provided in Section 3.1.

In this study, we present Bratter, an instruction set extension for supporting forward CFI solutions in RISC-V. Similarly to existing works, Bratter also provides special instructions to verify the behaviors of CTIs. Bratter introduces a *branch tag register* and provides special instructions that allow developers to set and verify the value of the register. However, unlike previous studies, the branch tag register supports *multiple 8-bit long branch tags*; thus, it can effectively support various CFI policies simultaneously. We implemented the prototype of Bratter on RISC-V, which is an open source instruction set architecture. To verify the feasibility of Bratter, we implemented well-known CFI policies (i.e., branch regulation and function-type based CFI) using Bratter. Our evaluation indicated that Bratter can efficiently support these CFI solutions.

## 2. Background

In this section, we provide background information for Bratter, such as jump instructions and the reserved elements of the RISC-V [11].

### 2.1. Control Transfer Instructions

There are two types of CTIs: direct and indirect. In RISC-V, beqz, bnqz, j and jal correspond to direct CTIs, whereas jalr and jr are indirect CTIs. Note that in the direct CTIs, the target address is obtained from the offset (±4 KiB ranges) embedded in the instruction encoding, whereas in the case of indirect CTIs, the target address is loaded from the general purpose register specified in the instruction encoding.

Consequently, attackers typically exploit indirect CTIs by tampering with the target address in the register, and they manipulate the control flow of the victim process to launch control-flow hijacking attacks such as ROP [12], JOP [13] and call-oriented programming (COP) [14]. Indirect CTIs can be categorized according to their expression in the CFG. Specifically, in CFG, indirect branches and indirect calls are called *forward-edges*, and a return instruction is called *backward-edge*. Consequently, CFI solutions can also be divided into forward-edge and backward-edge CFIs according to what they aim to protect.

### 2.2. Hints Instruction

RISC-V instruction set architecture (ISA) provides hint instructions, which are composed of instructions that do not require processing under particular circumstances. The hint instructions are treated as nop instructions when the behavior is not implemented. Table 1 shows the table of several examples of hint instructions with *constraints* and *code points*. The column *constraints* explains the conditions for the hint instruction. The column *code points* indicates the range of the value provided by the instruction. Moreover, these hint instructions can be divided into two ways: for future standard use, which means reserved for extending the ISA in the future, and for custom use, which means for user custom. For instance, when srli instruction has the register x0 as the rd field, srli can be used for the hint instruction. Because x0 is the zero register in RISC-V, the result will be discarded. Moreover, the developer can configure 10 bits of the srli instruction except for opcode and rd.

### 2.3. Control Status Register

In RISC-V, control status registers (CSRs) are special purpose registers that represent system configurations, such as MMU and architecture extensions. RISC-V provides a different set of CSRs to each privilege level (user, supervisor, machine). The higher privilege level instruction can access lower-level CSRs. However, the opposite is not true. Unlike general purpose registers, CSRs can only be accessed by special instructions, not by any arithmetic and memory instructions. RISC-V provides a 12-bit encoding space for CSRs, and among them, several CSR numbers are reserved for custom use.

## 3. Design and Implementation

In this section, we first explain the motivation and design goals, followed by the design and implementation of Bratter.

### 3.1. Problem Statement

**Figure 1:**
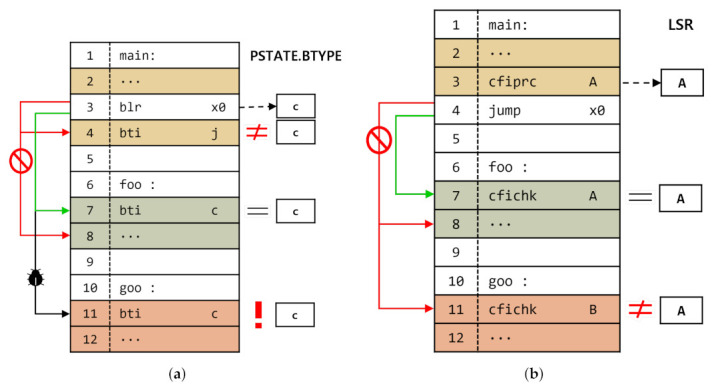
Simple example for ARM BTI [4] and SWT [6]. Assume that the indirect call in the main function is intended to invoke the foo function: (**a**) ARM BTI; and (**b**) SWT.

Recently, commercial processor architectures (ARM and Intel) announced special instruction set extensions, i.e., ARM BTI and indirect branch tracking (IBT), to defend against control-flow hijacking attacks. In Intel IBT, the instruction located at the target address of the branch instruction should be a special landing pad instruction, that is an endbranch. Otherwise, the processor generates a control protection fault. Similarly, ARM BTI also provides a special landing pad instruction, i.e., bti. However, in ARM BTI, developers can configure a branch type that can jump to each landing pad instruction by configuring the corresponding operand. For example, as shown in Figure 1a, an indirect call (i.e., blr x0) at line 3 in the *main* function can jump to line 7 and line 11, which are the entries of functions (i.e., *foo* and *goo*), as there is a landing pad instruction with operand ‘c,’ which means a call branch type. However, if the indirect call attempts to execute the code that begins with bti j, such as in line 4, the processor would generate a fault (i.e., *branch target exception*) because operand ‘j’ means the jump branch type. In addition, when an indirect control transfer occurred, targeted to a not-bti instruction, such as in line 8, the processor also generates a fault. Consequently, by using these extensions, developers can reinforce the program against control-flow hijacking attacks. However, according to our preliminary analysis, these extensions are not capable of implementing sophisticated defense solutions. For example, in Figure 1a, the functions *foo* and *goo* have the same operand c, and the indirect call instruction in the *main* (line 3) can branch to both functions. In other words, because they do not distinguish between landing pad instructions with the same branch types, an attacker can exploit the indirect call instruction to execute arbitrary functions, that is, a function reuse attack [15].

Meanwhile, some researchers have proposed an extension that can define multiple rules, unlike ARM BTI and Intel CET. Figure 1b shows the application of the extension to ensure the control-flow integrity of indirect calls. This extension provides a value for each function and stores it in the dedicated register (i.e., label state register (LSR)) before indirect control-flow transfer (line 3). Subsequently, the entry point of the function compares the given function signature value with the value stored in the register (line 7). It provides control-flow integrity more precisely than ARM BTI because the signature value is stricter than the jump types. For example, line 4 can jump to the function foo; however, it cannot jump to the function goo, unlike ARM BTI.

**Figure 2:**
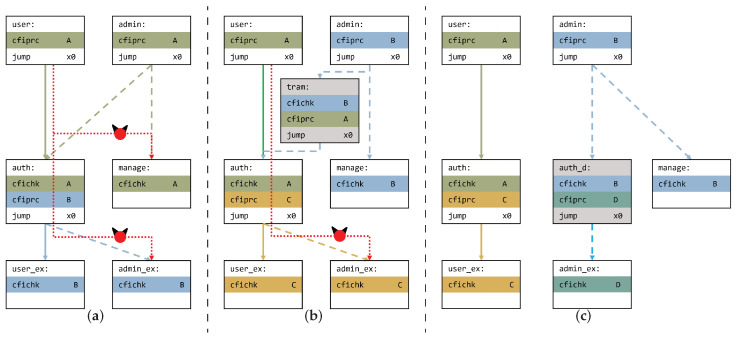
Control-flow sensitive example. Each line style represents a different path level. The solid shows user-level and the dashed shows admin-level. The red dotted line means unintended malicious paths: (**a**) problem state; (**b**) trampoline; and (**c**) duplication.

However, this extension also has some limitations in terms of programmability and security. For example, we assume a multi-level system that has a context-sensitive path as shown in Figure 2a. This must distinguish between paths from *user* to *user-ex* and *admin* to *admin-ex* that pass through *auth* function. Moreover, *management* function can only be executed using the *admin* function. *auth* function can be executed both by the *user* and *admin*; therefore, they need the same tag value, that is, A. Thus, *user* function can execute the *manage* function because the *user* and the *admin* have the same tag value. Furthermore, the *auth* function can invoke both *_ex* functions. From this point, both functions need to have the same tag value, that is, B. Therefore, the *user* can execute the *admin_ex* function through *auth* function. These unintended paths are represented by dotted lines in Figure 2a.

Figure 2b uses a trampoline to solve this problem; In the *tram* function, it checks the tag for the *admin* function and sets a tag that will be checked in *auth* function. Therefore, the *user* cannot invoke the manage function because *admin* and *user* have different tag values. However, the *user* can still invoke *admin_ex* because both *_ex* functions have the same tag value while they are both invoked by *auth* function.

Then, Figure 2c shows a solution in which the *auth* function is duplicated for *user* and *admin*. By having a relationship with the *admin* function, a tag value B is demanded; at this point, *auth* for *admin*, that is, *auth_d*, demands B and sets the tag value C for *admin_ex*. Therefore, the *user* cannot invoke the *auth_d* function because the *user* function has a tag value A, which means that *user* can only execute the *auth* function. However, this approach wastes memory space for the same function duplicated differently, for only one signature value.

### 3.2. Design Goals

We designed Bratter with the following design goals.

**G1. Programmability.** In existing CFI studies, the legitimacy of changing the control flow is evaluated based on various criteria such as function signature [3] and code range [16]. Therefore, we designed Bratter as a flexible instruction-set extension to support such CFI policies.

**G2. Security.**Bratter is an instruction set extension similar to other extensions, such as cryptography. Thus, to enforce the control flow integrity of the target program, developers should develop their solutions using Bratter instructions. However, if Bratter instructions can be exploited by an attacker, the security guarantee of the solution would be neutralized. Thus, we designed Bratter to be safe from such software exploitation.

**G3. Compatibility.** We designed a Bratter to minimize changes to the existing processor architecture. Otherwise, Bratter cannot be a widely used extension.

### 3.3. Overview

**Figure 3:**
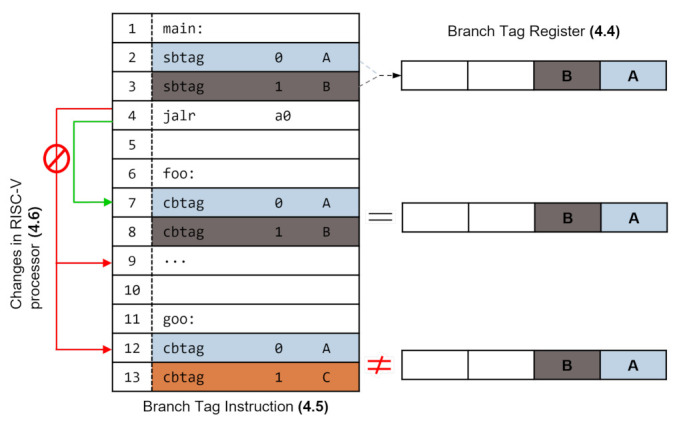
Overview of the Bratter architecture and behavior model.

To check the legitimacy of control transfers, Bratter introduced a branch tag into the system. Specifically, as shown in Figure 3, Bratter is composed of the following: *branch tag register* (BTR), *Bratter* *instructions* and *branch tag mismatch exception*. The BTR is a CSR register dedicated to holding branch tag values. Bratter provides special instructions, sbtag, i.e., set branch tag register, and cbtag, i.e., check branch tag register, to manage the branch tag values in the BTR. Specifically, sbtag is an instruction to assign a tag value to BTR, and cbtag is an instruction to check whether this value is legitimate. The detailed implementation and operation of the BTR and Bratter instructions are explained in Section 3.4 and Section 3.5. To detect control-flow hijacking attacks that violate the security policy of Bratter, we added a new exception to the RISC-V architecture, described in Section 3.6.

### 3.4. Branch Tag Register

**Figure 4:**
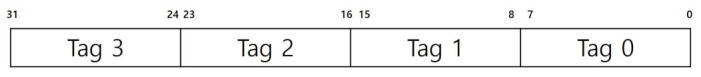
Encoding of the *branch tag register*.

In Bratter, BTR is the storage for branch tags. As shown in Figure 4, the BTR is a 32-bit register and it has four fields for four 8-bit branch tag values (tag–tag3). In the current implementation of Bratter, there is a single BTR in the system. However, the number of BTR can be increased depending on the required number of branch tag values. The changes in Bratter when there are multiple BTR are described in Section 7. For software compatibility (**G1**) and security (**G2**), we implemented the BTR as a reserved CSR in the RISC-V architecture. In other words, should we reserve one of the general-purpose registers as BTR, it will not be compatible with the software that uses the reserved register; however, in Bratter, we are able to obtain software compatibility by using the unused CSR. Furthermore, unlike general purpose registers, CSR can only be accessed through special instructions (see Section 3.5), preventing unintended access to the BTR.

### 3.5. Bratter Instructions

**Figure 5:**
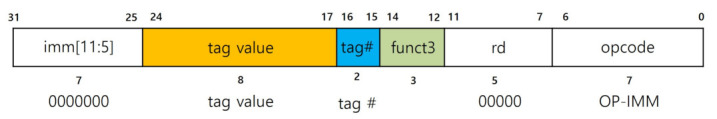
Encoding of Bratter instructions.

To enforce CFI using branch tag values, developers should be able to assign values to the branch tags and check their validity. For this, Bratter provides two dedicated instructions, sbtag and cbtag. These instructions take two operands, 2-bit *id* and 8-bit *value*. As a consequence, sbtag assigns a *value* to the *id*-th branch tag and cbtag checks whether the assigned value is the same. We added two dedicated instructions, that is, sbtag and cbtag, to access the BTR. We implemented these by extending slli and srli instructions because these are hint instructions when the rd field is x0 and provide 10 bits of code points, which we need. Owing to this feature, Bratter instructions are performed when the rd field is x0. Furthermore, we provide compatibility with legacy machines because it behaves like nop when the Bratter is not adopted. Before the control transfer (e.g., indirect jump, indirect call), we inserted the sbtag instructions to store the tag value into the BTR. After the control transfer, the cbtag instruction guarantees CFI by using the tag value and tag number. Figure 5 depicts the encoding form of the instruction, which consists of six fields. The form is almost the same as that of the base instruction. However, the rd field is x0 as we extend the hint instruction. Then, we use 10-bits of code points for taking the two operands, the *value* and the *id*.

**Figure 6:**
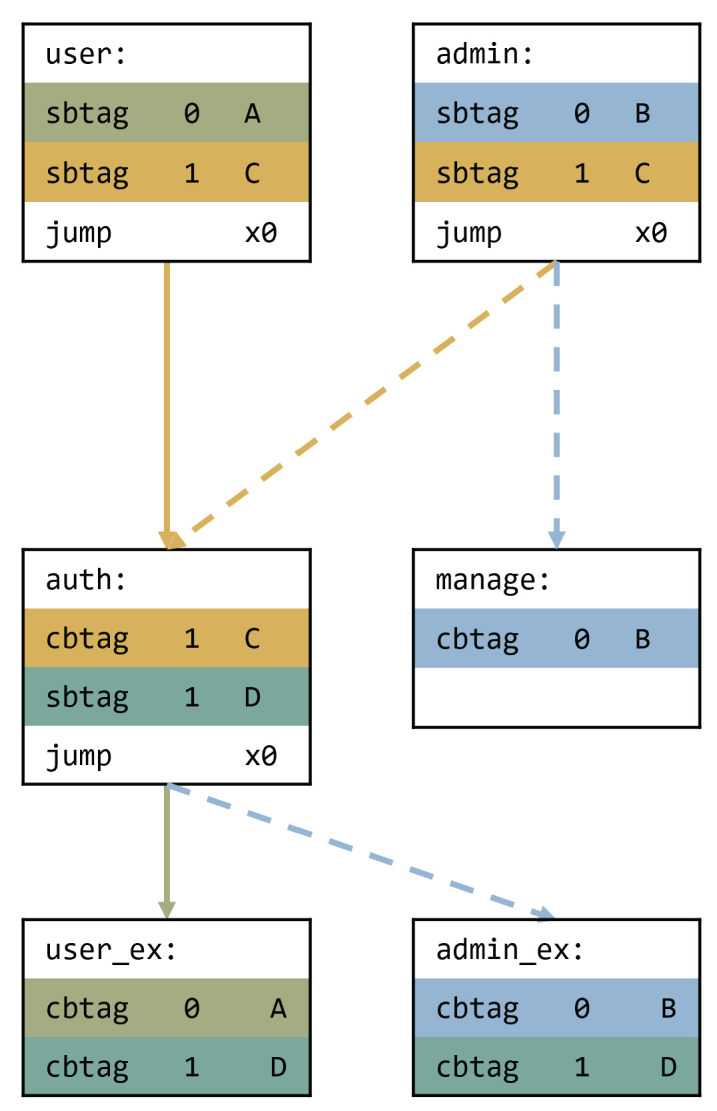
Address the problem in Figure 2a by using Bratter.

Figure 6 shows that our approach can solve the problem. Let us describe the aspects of the user function. First, we set the tag value A in tag0 at the user function and C in tag1. Therefore, the auth function can check tag1 with value C to verify the legitimate functions (i.e., user and admin) and set tag value D in tag1. Then, user_ex is invoked, and it checks tag value A with tag0 and D with tag1. Owing to multiple fields, both ex functions check the two tag values (A, D) and (B, D). Therefore, the user function cannot invoke functions exclusive to the admin.

### 3.6. Changes in RISC-V Processor

To ensure CFI, the processor needs to guarantee cbtag instruction after the control transfer. This means that after the control transfer, when the next instruction is not cbtag, the processor should halt the process and an exception occurs. For this, we leverage the internal counter; furthermore, when the tag mismatch occurs, we raise the same exception, and halt the process. The best approach to address the exception is by adding a new exception to the architecture. However, it is challenging to implement and the available exception numbers are difficult to find. Therefore, we use a similar exception: illegal instruction. The exception will be realized in future work when it is implemented in hardware.

## 4. Use Cases

To show the programmability of Bratter (G1), we implemented two well-known CFI solutions, *function signature check* and *branch regulation*, using Bratter.

### 4.1. Function Signature Check

The function signature check [3] is a solution to prevent an attack that exploits indirect calls. Specifically, it verifies the behavior of indirect calls by checking whether the target function has a valid function signature. For this, this solution calculates the signature values for all functions in the program by analyzing the source code or program binary code. The function signature is typically derived from the number of parameters and the data type of the return value and parameters. Then, it inserts instructions before indirect calls to check whether the called function has a legitimate signature of the indirect call. If the signature of the called functions is not same as the legitimate one for the indirect call, then the program execution is halted or an exception arises.

**Figure 7:**
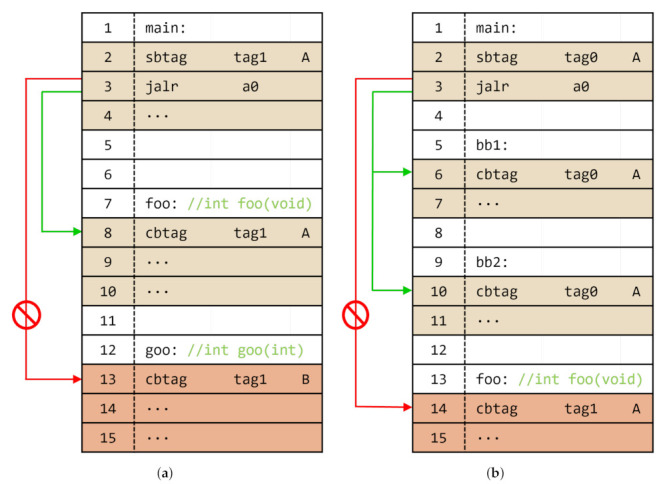
CFI solutions using Bratter: (**a**) indirect call protection; and (**b**) indirect jump protection.

To implement this solution using Bratter, we insert instructions to the target function entries and before indirect calls. Specifically, the sbtag instruction is inserted before the indirect call, and the cbtag instruction is inserted at the first function entry point (note that some functions can be called not only by indirect calls but also by direct calls; in this case, for compatibility, we also insert sbtag instructions before those direct calls). For example, as shown in Figure 7a, the indirect call in line 3 is written to call any function that has an integer return value and no argument. For this, in line 2, the instruction is inserted to calculate the signature value for line 8. Subsequently, line 8 shows the instruction that verifies the signature value and tag number. As each function (foo and goo) has a different argument, line 3 cannot call line 13.

### 4.2. Branch Regulation

Branch regulation [16] prevents attacks that exploit indirect branches, such as indirect calls, indirect jumps and returns. Specifically, it enforces the branch to target only the entry point of the function or the basic block. However, the solution of the indirect call is provided in Section 4.1; moreover, as the return is not the focus of our study, we focus on the indirect jump case. Branch regulation also analyzes the source code or program binary. However, rather than calculating the signature values, it only calculates the jump boundaries within the function and the right target of the jump, that is, the entry point of a basic block. In other words, the indirect jump can only target the basic block entry point within a function where the jump instruction locates. Therefore, it provides the same block ID to each basic block in a function.

To implement the branch regulation by using Bratter, we insert instructions to the basic block entries and before the indirect jumps. For example, as shown in Figure 7b, the indirect jump in line 3 is written to jump any basic block in the function main. Therefore, all the basic blocks within the function main have the same block ID, that is, A, and the same tag number, that is, tag0. From this point, the indirect jump in line 3 can only target *bb1* and *bb2* because they have a legitimate signature value and tag number at the entry point. Furthermore, a fault will occur when targeted out of bound, for example, out of the main function such as foo in line 14, because it has an illegitimate block ID or tag number.

## 5. Evaluation

### 5.1. Experimental Setup

To evaluate Bratter based on use cases in Section 4, we extended the LLVM [17] compiler framework. In detail, we made a custom machine instruction level pass that inserts sbtag and cbtag instructions according to the security solutions described in Section 4. This machine instruction level pass runs on all functions and each basic block right after the IR is converted to machine IR. We utilized the machine instruction builder interface (i.e., BuildMI()) provided by LLVM to insert Bratter instructions.

We implemented Bratter on top of a spike RISC-V ISA simulator [18] and used the Beebs benchmark suite [19] to measure the code size and performance overheads when security solutions in Section 4 are applied in benchmark program. *Bratter_FS* and *Bratter_BR* denote the cases in which the function signature check approach and the branch regulation approach are applied, respectively. *Bratter_Both* shows the result that two approaches are simultaneously adopted. The code size was measured using riscv-gnu-toolchain [20]. The execution time was obtained by logging the executed instructions in sequence and by multiplying the weighted cycle with each instruction.

### 5.2. Code Size Overhead

**Figure 8:**
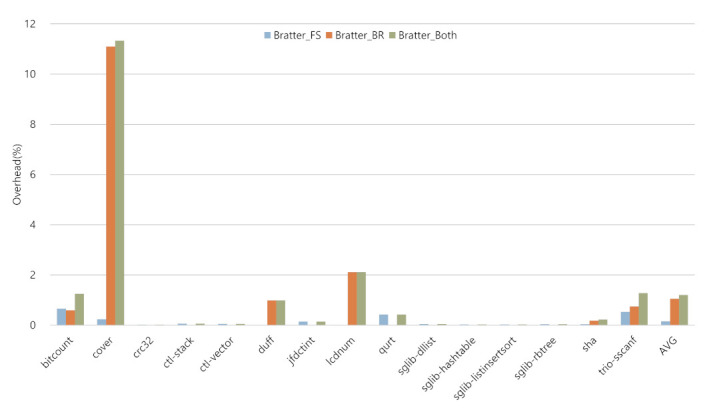
Code size overhead of Bratter.

The code size overhead of Bratter is shown in Figure 8. The Bratter_FS case shows an average code size overhead of 0.15%, whereas the Bratter_BR and Bratter_Both cases show average overheads of 1.05% and 1.20%, respectively.

The Bratter_FS case showed a code size overhead ranging from 0% to 0.66%. In the normal Bratter_FS case, sbtag and cbtag are pairing as caller and callee, respectively. From this feature, the term *bitcount* shows the most significant overhead in the Bratter_FS case and has the most numerous caller–callee pairs. On the other hand, *trio-sscanf* has a data structure with function pointers, and sbtag (48) and cbtag (3) are not paired. Furthermore, although it has the most numerous added instructions, it does not show the most significant code size overhead since the added instruction size is very small compared to the original code size.

In Figure 8, the Bratter_BR case showed a code size overhead ranging from 0% to 11.10%. In this case, the main factor causing overhead is the number of candidate blocks that can be a target of indirect jumps. From this feature, term *cover* represents the most significant code size overhead. As represented in Table 2, *cover* has just three sbtag; however, it also has 190 cbtag instructions. Furthermore, the basic blocks are constructed by three instructions, including CTI; from here, the added cbtag means 125% overhead to that basic block. After that, the Bratter_Both case shows an overhead of 0.02% to 11.33%, and this case represents the result of adding Bratter_FS and the Bratter_Both—since the Bratter_FS and Bratter_BR approaches are exclusive.

### 5.3. Execution Time Overhead

**Figure 9:**
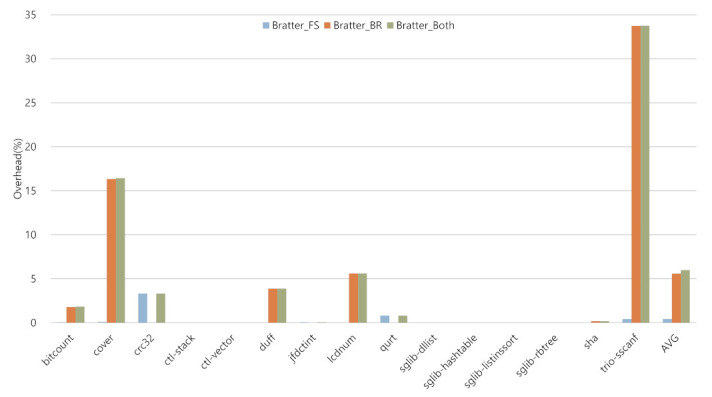
Execution time overhead of the Bratter.

The execution time overhead of Bratter is shown in Figure 9. The execution time overhead measurement was performed by logging the executed instruction histogram of the spike simulator and multiplying the weighted cycle represented in Table 3 to each instruction. We weighted two cycles for both instructions, i.e., sbtag and cbtag, considering the BTR register access. On average, Bratter_FS showed 0.43%, Bratter_BR and the Bratter_Both show 5.59% and 5.99% execution time overhead, respectively.

The Bratter_FS case showed an increase from 0% to 3.32%. The *sglib* families showed a code size overhead in Section 5.2 since they include a library with a function that has one or two indirect CTIs. Nevertheless, they showed 0% overhead in execution time because they did not invoke these functions in practice. After that, term *crc32* showed the most significant overhead in the Bratter_FS case. In this term, Bratter added four instructions; however, in a loop, it calls the function that includes cbtag with sbtag; therefore, each instruction is executed 2050 times.

The case Bratter_BR shows overheads from 0% to 33.74% and Bratter_Both shows overhead from 0% to 33.76%. In Bratter_BR case, the term *trio-sscanf* executed less added instructions than the Bratter_FS case; nevertheless, it shows the most significant overhead. Because, when in the Bratter_BR case, the *trio-sscanf* executed memory access instructions, e.g., st, ld, more than the Bratter_FS case. Subsequently, the term *sha* executed the most number of instructions in the Bratter_BR case; however, the original executed instruction count was enormous—so the execution time overhead was very low. The term *duff* executed more cbtag than sbtag because it has continuous basic blocks and each basic block can be a target of the indirect jump. Since the Bratter_BR case restricts the jump boundary into the function region, then each basic block in a function has the same tag values; thanks to this feature, the term *duff* does not violate the policy.

## 6. Related Work

Prior to Bratter, numerous studies have been conducted on CFI. In this section, we briefly explain how these studies relate to Bratter.

### 6.1. Software-Based Approach

Abadi et al. [2] first proposed a code-instrumentation-based CFI mechanism. They assigned a label ID to each valid target address and verified the legitimacy of the label ID before every call site. However, for such fine-grained CFI policies, the mechanism had to execute a series of additional memories and compare instructions; thus, it suffered from a high performance overhead. Later works [21,22,23] focused on addressing the performance issue by enforcing coarse-grained CFI policies. However, this strategy inevitably creates a security hole, and several studies have indicated that an attack is possible on coarse-grained CFI [24,25]. In contrast, because Bratter provides special instructions for CFI, developers can implement fine-grained CFI policies using these.

### 6.2. Instruction Set Extension for CFI

In the software-based approach, the main factor of the performance overhead is that whenever a control transfer occurs at runtime, they must perform a couple of instructions for checking CFI rules. Thus, some studies have proposed a special instruction set that can efficiently check the CFI rules. These works typically provide instructions that set the CFI rules before the control transfer instructions and check the CFI rules at the target address. However, as explained in Section 3.1, there is a problem in HCFI [8], SWT [6] and EXCEC [26]. In short, they have to add a trampoline code through complex code analysis to support context-sensitive CFI rules. ABCFI [10] relieves the need for the trampoline by introducing a slot containing legitimate target addresses. Concretely, they make the indirect branch instructions jump to one of the valid addresses in the slot, not just one target address. However, to allocate multiple slots, they have to align the program code, so they suffer from high code size overhead. In Bratter, unlike existing works, Bratter provides special storage (i.e., branch tags) that can hold multiple CFI rules at runtime, so Bratter can efficiently enforce context-sensitive CFI policies without trampolines and slots. HAFIX [7] enforces CFI through backward-edge protection based on labels for each function that are stored in a dedicated memory area; thus HAFIX is orthogonal with Bratter since Bratter is designed to enforce forward-edge CFI.

### 6.3. Trace-Based Approach

Several works have utilized the debug feature of the processor architecture such as ARM ETM [27,28,29], Intel PT [30,31,32,33,34,35] and RoCC [9,36]. They typically extract the control trace of the target process and verify whether an attack occurred through the trace. To efficiently address the tremendous amount of trace at runtime, some studies introduced a special co-processor [9,29,37,38] and they can alleviate the performance overhead to some degree. However, this approach cannot prevent the attack because it detects the attack after analyzing the trace in the separate hardware.

## 7. Discussion

Currently, the implementation of Bratter provides one 32-bit branch tag register (including four 8-bit branch tags). Furthermore, this configuration has been sufficient to cover two security solutions in our benchmark program, as shown in Table 2. However, in some programs, the number of this tag configuration cannot be enough to express complex CFI rules. To deal with this, Bratter can be extended to support multiple branch tag registers as follows. At first, we have to introduce additional branch tag registers in the system. According to the RISC-V instruction set manual, they provide encodings for 256 custom CSRs, so we believe that this number of CSRs is sufficient to support complex CFI rules. Then, we need to change the special instructions to manipulate branch tag registers. Because if the number of tags or the number of ids is increased, more bits are required to express the id–tag value pair. Fortunately, we found some hint instructions that provide more code points than srli and slli. For instance, slti and sltiu provide $2^{17}$ code points for custom use.

## 8. Conclusions

This paper presents Bratter, an instruction extension for the forward CFI on RISC-V. Specifically, Bratter introduces branch ID registers to store the identifiers of the branch instructions to be executed. Bratter also adds dedicated instructions to set these registers and verifies the control-flow integrity based on them. The evaluation results confirm that Bratter can support CFI solutions in an efficient and scalable manner.

## Figures and Tables

**Table 1:** Hint instruction table in RISC-V. The first column presents the base instruction. The second shows the constraints’ conditions. The third shows the number of possible expressions with programmable bits in each instruction. The last presents the purpose of the hint instruction.

Instructions	Constraints	Code Points	Purpose
LUI	rd = x0	$2^{20}$	Reserved for future standard use
AND	rd = x0	$2^{10}$	

SLLI	rd = x0	$2^{10}$	Reserved for custom use
SRLI	rd = x0	$2^{10}$	

**Table 2:** The number of added and executed instructions table during Bratter benchmark.

Benchmark	the # of Added Instructions				the # of Executed Instructions			
	Bratter_FS		Bratter_BR		Bratter_FS		Bratter_BR	
	sbtag	cbtag	sbtag	cbtag	sbtag	cbtag	sbtag	cbtag
bitcount	5	5	1	8	4	4	160	160
cover	2	2	3	190	2	2	360	360
crc32	2	2	0	0	2050	2050	0	0
ctl-stack	1	1	0	0	2	2	0	0
ctl-vector	1	1	0	0	2	2	0	0
duff	0	0	1	8	0	0	2	76
jfdctint	1	1	0	0	2	2	0	0
lcdnum	0	0	1	16	0	0	10	10
qurt	3	1	0	0	6	6	0	0
sglib-dllist	2	0	0	0	0	0	0	0
sglib-hashtable	1	0	0	0	0	0	0	0
sglib-listinssort	1	0	0	0	0	0	0	0
sglib-rbtree	2	0	0	0	0	0	0	0
sha	2	2	2	16	2	2	3070	3070
trio-sscanf	48	3	7	65	80	80	64	64

**Table 3:** Weighted cycles for measuring the Bratter execution time overhead.

Instruction	sbtag	cbtag	slli	srli	ld	st	div	reg	ecall	others
Cycle	2	2	2	2	3	2	1	1	10	1

## Data Availability

Data for the experiments are available from the authors on request.
